# First-in-human pilot study of snapshot multispectral endoscopy for early detection of Barrett’s-related neoplasia

**DOI:** 10.1117/1.JBO.26.10.106002

**Published:** 2021-10-09

**Authors:** Dale J. Waterhouse, Sophia Bano, Wladyslaw Januszewicz, Dan Stoyanov, Rebecca C. Fitzgerald, Massimiliano di Pietro, Sarah E. Bohndiek

**Affiliations:** a University of Cambridge, Department of Physics and CRUK Cambridge Institute, Cambridge, United Kingdom; b University College London, Wellcome/EPSRC Centre for Interventional and Surgical Sciences, London, United Kingdom; c Medical Centre for Postgraduate Education, Department of Gastroenterology, Hepatology and Clinical Oncology, Warsaw, Poland; d University of Cambridge, MRC Cancer Unit, Hutchison/MRC Research Centre, Cambridge, United Kingdom

**Keywords:** multispectral, endoscopy, dysplasia, computer assisted diagnosis, esophagus

## Abstract

**Significance:** The early detection of dysplasia in patients with Barrett’s esophagus could improve outcomes by enabling curative intervention; however, dysplasia is often inconspicuous using conventional white-light endoscopy.

**Aim:** We sought to determine whether multispectral imaging (MSI) could be applied in endoscopy to improve detection of dysplasia in the upper gastrointestinal (GI) tract.

**Approach:** We used a commercial fiberscope to relay imaging data from within the upper GI tract to a snapshot MSI camera capable of collecting data from nine spectral bands. The system was deployed in a pilot clinical study of 20 patients (ClinicalTrials.gov NCT03388047) to capture 727 *in vivo* image cubes matched with gold-standard diagnosis from histopathology. We compared the performance of seven learning-based methods for data classification, including linear discriminant analysis, k-nearest neighbor classification, and a neural network.

**Results:** Validation of our approach using a Macbeth color chart achieved an image-based classification accuracy of 96.5%. Although our patient cohort showed significant intra- and interpatient variance, we were able to resolve disease-specific contributions to the recorded MSI data. In classification, a combined principal component analysis and k-nearest-neighbor approach performed best, achieving accuracies of 95.8%, 90.7%, and 76.1%, respectively, for squamous, non-dysplastic Barrett’s esophagus and neoplasia based on majority decisions per-image.

**Conclusions:** MSI shows promise for disease classification in Barrett’s esophagus and merits further investigation as a tool in high-definition “chip-on-tip” endoscopes.

## 1 Introduction

Patients with Barrett’s esophagus^1^ (BE) undergo routine surveillance using high-resolution white light endoscopy (HR-WLE) and random biopsies to detect the presence of dysplasia, which increases the risk of developing esophageal adenocarcinoma.^2^ Early detection of the precursor dysplastic lesions, or early-stage cancer, enables curative intervention, increasing the 5-year survival rate from just 15%–25% to 80%.^3^^–^^5^ Unfortunately, these precursor lesions can be challenging to identify on standard-of-care HR-WLE.^6^^,^^7^

Advanced optical imaging modalities have potential to impact patient care.^8^ With demand for endoscopy predicted to rise substantially over the next decade,^9^ the unmet clinical need for optical methods with improved diagnostic yield and/or lower cost per procedure is particularly acute. When light travels through tissue, it is absorbed by endogenous chromophores, such as hemoglobin, and scattered by endogenous structures, such as cell nuclei.^10^ Disease-related structural and biochemical changes in the epithelial layer of the gastrointestinal (GI) tract can alter the distribution and abundance of these absorbers and scatterers—for example, neovascularization increasing hemoglobin abundance in the epithelium^11^—resulting in subtle wavelength-dependent changes in reflected light, which can be measured not only by point-based spectroscopy methods^12^ but also by hyperspectral imaging methods that capture spatially resolved (x,y) and spectral (wavelength, λ) information in a single data set, often using mechanical scanning.^13^^,^^14^

Multispectral imaging (MSI) represents a compromise between the extremes of point-spectroscopy and hyperspectral imaging, providing typically up to 10 wavelengths of information, which can be sufficient to resolve color features, while requiring generally simpler optics and enabling faster scan times. HR-WLE actually represents a simple case of MSI, where tissue features are measured in three broad color bands: red (620±40  nm), green (540±40  nm), and blue (470±40  nm) to replicate the spectral sensitivity of human vision.^15^ Though this represents a substantial improvement over monochrome imaging, finer spectral information is lost as the light is pooled into three broad bands. More recently, narrow band imaging^16^ was developed specifically to enhance contrast for vasculature and superficial mucosal morphology, using two narrow illumination bands (415±10 and 540±10  nm). Similarly, blue-light imaging uses a narrow band of blue light (410±10  nm) to improve the contrast of vessels and mucosal pits.^17^ In combination with data analysis using spectral unmixing algorithms, multispectral and hyperspectral imaging have been used in a range of biomedical applications to visualize the vascular pattern and the oxygenation status of blood,^18^^–^^26^ to improve detection of gastric^27^ and colorectal lesions,^28^^–^^30^ for intraoperative image guidance,^31^ to identify residual tumor,^32^ and to perform tissue segmentation.^33^^,^^34^

The majority of MSI devices fall into two categories: amplitude-division, where the light beam is divided into two new beams, and field-division, where the light is filtered or divided based on its position in the beam.^35^ With all MSI approaches, a trade-off among spatial, spectral, and temporal resolution must be considered alongside cost, complexity, size, and robustness. Previously reported spectral endoscopy systems generally use amplitude-division, including multiple bandpass filters,^29^^,^^36^ tunable filters,^28^^,^^37^ laser lines,^38^^–^^40^ or detectors dedicated to separate spectral bands.^38^^,^^39^ These amplitude-division systems are typically bulky, costly, and more susceptible to misalignment in a clinical environment. Furthermore, they often require sequential acquisition resulting in slow acquisition rates, unsuitable for real-time clinical imaging. Several field-division approaches are available, including line-scanning^41^ and image mapping spectroscopy.^20^ Spectrally resolved detector arrays (SRDAs) are a relatively new addition,^18^^,^^42^^,^^43^ exploiting spectral filters deposited directly onto the imaging detector in a mosaic pattern to achieve a low-cost, compact, and robust device that is well suited to clinical application.

Here, we sought to determine whether MSI using an SRDA could be applied in endoscopy to improve detection of dysplasia in the upper GI tract in patients with BE. We created a custom MSI endoscope and undertook a first-in-human pilot clinical study to acquire *in vivo* MSI data from esophageal tissue matched with gold-standard histopathological diagnosis of disease state. These data were then subjected to machine learning-based classification methods, indicating that despite substantial intra- and interpatient variations, MSI has the potential to resolve different esophageal disease states in patients with BE.

## 2 Methods

### 2.1 Snapshot Multispectral Endoscope for *In Vivo* Clinical Imaging

**Fig. 1:**
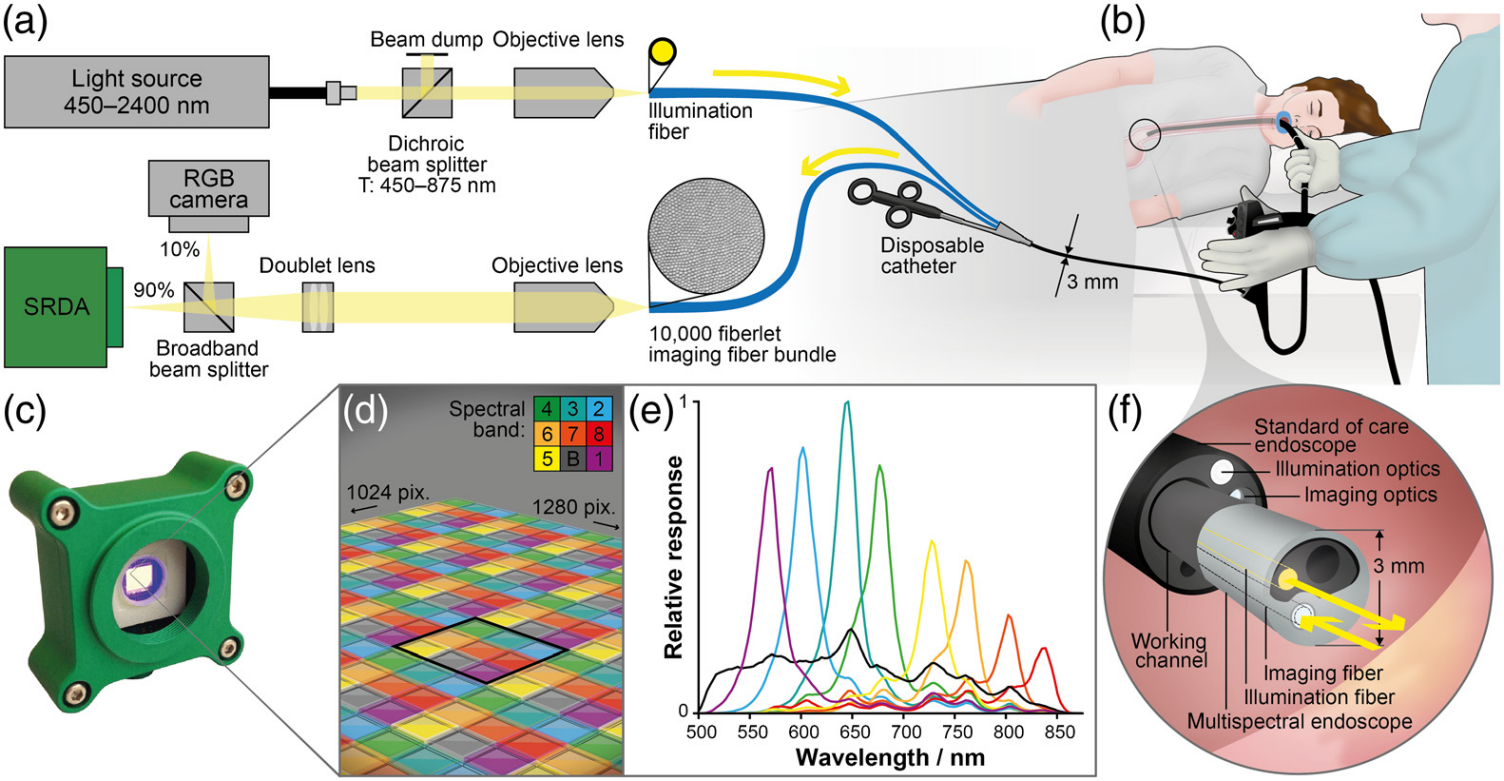
A compact snapshot multispectral endoscope for esophageal imaging. (a) Light from a broadband supercontinuum light source enters the patient via a single illumination fiber. Reflected light is returned via a 10,000 fiberlet imaging bundle. (b) The “babyscope” enters the esophagus via the working channel of a standard of care endoscope. (c) Light is detected by an SRDA. (d) This includes a 3×3 mosaic of color filters, eight narrow bands, and one broad band (B) deposited directly on the sensor pixel. (e) The spectral response of the detection channel of the multispectral endoscope is shown. (f) Light is delivered to the esophageal lumen via the babyscope illumination fiber and diffusely reflected light is collected by the imaging fiber bundle.

In the future, SRDAs could be deployed as “chip-on-tip” cameras at the distal end of an endoscope, however, such integration requires significant miniaturization of optics and changing a clinically approved device, which substantially extends the timeline required for first-in-human testing. Furthermore, the spectral properties of esophageal tissue are not well characterized nor reliably measured using *ex vivo* tissue.^44^ Thus, to facilitate clinical testing, we combined the SRDA with a CE-marked “babyscope” (PolyScope, PolyDiagnost, Germany), a small fiber-bundle-based endoscope that can be inserted through the accessory channel of a standard endoscope to relay light between external optics and the esophageal lumen. We have previously shown that SRDAs can be implemented in combination with such an imaging-fiber bundle without reducing resolution, since the resolution of fiberscope-based imaging is limited by the size of individual fiberlets rather than by the sensor resolution.^45^ The babyscope system included: a single fiber to relay illumination from outside the patient and direct it onto the tissue; and a 10,000-fiberlet imaging bundle to relay diffusely reflected light from the esophageal tissue to custom external detection optics [Fig. 1(a)]. The 3 mm diameter of this babyscope allowed it to be introduced through the accessory channel of a therapeutic gastroscope [Fig. 1(b)]. Utilizing this unmodified CE-marked device to relay light between the esophagus and external optics facilitated clinical application of the spectral endoscope.

At the proximal end of the babyscope, the illumination fiber was coupled to a broadband supercontinuum light source (SuperK COMPACT, NKT Photonics, United Kingdom), which provided illumination from 450 to 875 nm (Fig. S1 in the Supplementary Material). The proximal end of the imaging fiber was imaged and magnified using an objective lens (UPLFLN20x, Olympus, Japan). The light was split using a 90:10 plate beam splitter (BSN10R, Thorlabs, Germany) and focused using an achromatic doublet lens (f=100  mm, ACA254-100-A, Thorlabs, Germany); 10% of the light was focused onto a red–green–blue (RGB) camera (Grasshopper 3.0, IDS, Germany) to record reference images and the remaining 90% was focused onto the SRDA (CMS-V, SILIOS, France) [Fig. 1(c)]. The SRDA consists of nine spectral filters deposited as a 3×3 super-pixel [Fig. 1(d)] across a complementary metal oxide semiconductor sensor (NIR Ruby sensor, UI1242LE-NIR, IDS, square pixel size 5.3  μm matched to spectral filter size). The nine spectral filters comprise eight narrow bands of average full-width half-maximum 30 nm with center wavelengths 553, 587, 629, 665, 714, 749, 791, 829 nm, along with 1 broad band; 500 to 850 nm [Fig. 1(e)]. The optics were securely housed inside a light tight enclosure and mounted on an optical breadboard (MB4545/M, Thorlabs, Germany), which was fixed to a stainless-steel trolley (FW2901-3, Freeway Medical, United Kingdom) with a footprint of 512  mm×480  mm, allowing the system to be easily and safely transported in a busy and often crowded clinical setting.

This distal end of the multispectral endoscope accessed the esophagus via the working channel of a therapeutic gastroscope, directed by the articulation of the gastroscope to collect diffuse reflectance images alongside standard-of-care imaging [Fig. 1(f)], thus causing minimal disruption to clinical workflow.

**Fig. 2:**
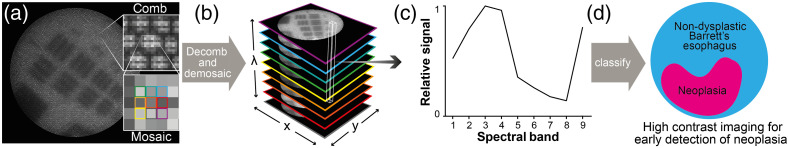
Data processing for snapshot multispectral endoscopy. (a) Raw collected data contain comb and mosaic artifacts from the imaging fiber and SRDA, respectively. (b) These are removed by a demosaicing and decombing algorithm to produce a spectral data cube. (c) For a given pixel in the image cube, a nine-band per-pixel spectrum can be determined. (d) Using these per-pixel spectra, machine-learning-based classification algorithms can interpret the image cube to generate a classification map, thus helping to guide the endoscopist.

Endoscope settings were controlled using an interface developed in LabVIEW (National Instruments) running on a PC and a tablet (Surface Pro, Microsoft). Images captured by the SRDA were saved as 8-bit 1280×1024-pixel bitmap image files (to enable fast acquisition and immediate review of the images). These images contain two artifacts: a comb structure due to the imaging fiber bundle and a mosaic pattern due to the spectral filters of the SRDA [Fig. 2(a)]. For analysis, these were removed in a process of demosaicing and decombing as described previously,^45^ resulting in a 1280  pixel×1024  pixel×9 band image cube [Fig. 2(b)] with a spatial resolution of 240±20  μm at a working distance of 1 cm. For real-time display, three of the nine bands were decombed and displayed as a false-RGB image at up to 15 fps. For analysis, columns of the image cube can be treated as nine-point “per-pixel spectra” [Fig. 2(c)]. As high-dimensional image data would be challenging for an operating clinician to interpret in real-time, machine-vision techniques can be used to classify per-pixel spectra to produce a tissue map for endoscopic image guidance [Fig. 2(d)].

### 2.2 Color Chart Imaging

**Fig. 3:**
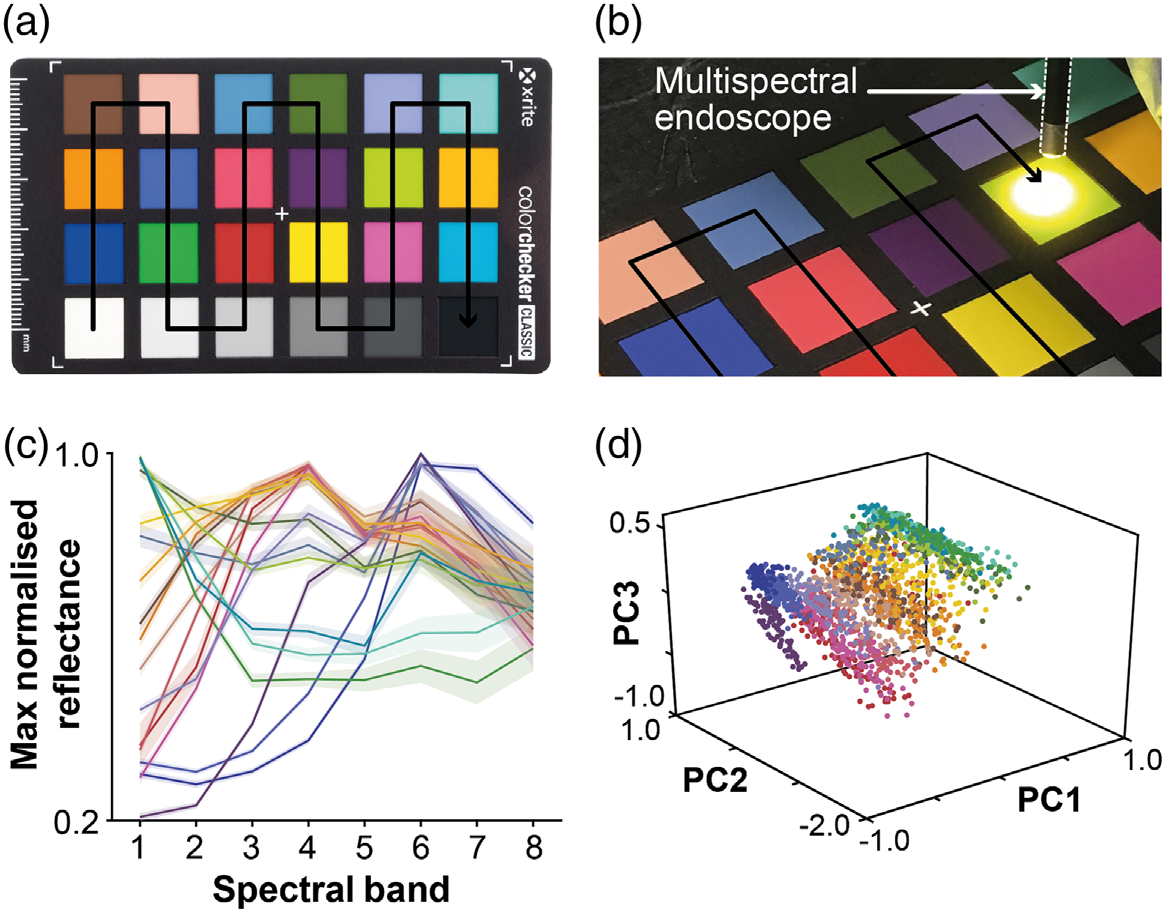
Color chart imaging. (a) A Macbeth color chart was used to validate the multispectral endoscope. (b) Handheld imaging of the Macbeth color chart took place prior to four of the *in vivo* trials. (c) The mean per-pixel spectra for each color on the Macbeth color chart are distinct, but overlap within their variation. Shaded region shown is standard deviation/5 for clarity. (d) PCA not only shows clustering of the per-pixel spectra by color but also shows the overlap of similar colors.

To test the accuracy of the snapshot multispectral endoscope in a controlled setting with known reference data, we performed handheld imaging of a Macbeth color chart (ColorChecker Classic Mini, x-rite) prior to four of the *in vivo* trials (Fig. 3). The color calibration target consists of 24 squares of painted samples, 18 of which contain colors whose spectral reflectance is intended to mimic those found in natural objects, and 6 of which are a uniform gray lightness scale.

### 2.3 First-in-Human Pilot Study of Snapshot Multispectral Endoscopy

The snapshot multispectral endoscope was deployed in a pilot clinical study to acquire *in vivo* images. This prospective pilot cohort study was carried out at the Cambridge Clinical Research Centre, Cambridge University Hospitals, United Kingdom. Eligible patients were adults (at least 18 years old) with a previous diagnosis of BE with an endoscopic length of at least 2 cm if circumferential (circumferential extent C≥2 according to the Prague classification) or at least 3 cm if not circumferential (maximal extent M≥3 according to the Prague classification). Exclusion criteria are given in the Supplementary Material. The trial was reviewed by Cambridgeshire Research Ethics Committee and was approved in March 2018 (18/NW/0134). The trial was registered at ClinicalTrials.gov (NCT03388047).

### 2.4 Endoscopic Procedure and Histopathology

The clinical procedure undertaken is summarized in Fig. S2 in the Supplementary Material. Each procedure was performed by a single experienced endoscopist (MdP).

i. After local anesthesia and/or conscious sedation (midazolam ± fentanyl), the patient was intubated with HR-WLE (H290Z, Olympus, Japan). After cleaning the esophageal mucosa using a water jet, the endoscopist thoroughly inspected the mucosal surface of the esophagus. Cautery marks were placed around two regions of interest: one suspicious lesion and one region of inconspicuous BE as a control. In the absence of suspicious lesions, one or more random areas within the BE segment were selected depending on the length of the BE. The HR-WLE video stream was recorded using a recording unit (SMP300, Extron).
ii. The endoscopist inserted the snapshot multispectral endoscope through the working channel of a therapeutic endoscope (GIF2T240, Olympus, Japan). The use of the therapeutic scope was required as its accessory channel was sufficiently large (3.7 mm diameter) to allow easy insertion of the multispectral endoscope (3.0 mm diameter at tip and 2.65 mm along length). The multispectral endoscope was used to image the regions of interest cautery marked in step (i). An endoscope cap was not used, as it can easily come into contact with lesions and induce contact bleeding. For the second half of the trial (trial number 11 onward), an additional control region of distant squamous tissue was inspected with the multispectral endoscope following inspection of the two marked regions.
iii. The multispectral endoscope was withdrawn, and the endoscopist proceeded to an endoscopic mucosal resection or biopsy (depending on the endoscopic findings and the level of suspicion). Control areas received biopsies only. Pathological assessment of biopsies was performed by an expert GI pathologist with extensive experience in reporting BE-related neoplasia.^46^^–^^48^ Histopathology was interpreted according to the revised Vienna classification.^49^ In the case of dysplasia, a second pathologist reviewed the diagnosis to achieve consensus.

For analysis, the most advanced disease present in each biopsy determined the label for the region such that three classes of spectra were acquired: (1) normal squamous; (2) nondysplastic BE (NDBE); and (3) neoplasia (consisting of dysplasia and intramucosal carcinoma).

### 2.5 Coregistration of Multispectral Images with Diagnosis from Histopathology

To enable matching of multispectral image data with the diagnosis from histopathology, the HR-WLE video stream and the video stream from the multispectral endoscope were synchronized and placed side-by-side for analysis. The HR-WLE video was carefully inspected to extract frames where the multispectral endoscope was sampling the marked regions of interest that were biopsied to give gold-standard diagnosis from histopathology. Since the esophageal lumen is quite featureless on MSI, the frames were identified using the cautery marks and other visible landmarks in the HR-WLE video stream to estimate the field of view of the multispectral endoscope. The tip of the multispectral endoscope was visible within the field of view of the HR-WLE endoscope, which facilitated identification of appropriate multispectral images that could be related to the gold-standard diagnosis made by histopathology.

### 2.6 Spectral Data Processing

Briefly, raw images were checked for saturation (pixel values>250), dark subtracted then decombed, to account for the structure introduced to the image by the individual fiberlets, and demosaiced, to separate the nine spectral bands, creating an image cube as outlined previously.^45^ In the image cube, low signal pixels were removed (max per-pixel spectrum value<50) as they are more likely to be affected by noise. Finally, each per-pixel spectrum was divided by a white light reference spectrum and normalized to the maximum of the per-pixel spectrum, such that the per-pixel spectra represent max-normalized reflectance. The white reference spectrum was an average of images captured from a white reflectance standard prior to each procedure to avoid variations introduced depending on experimental conditions.

For classification, the spatial dimensions were binned into 32×32  pixels as a compromise between retaining spatial information and increasing classification accuracy by reducing noise (Fig. S3 in the Supplementary Material). This resulted in 40-pixel×32-pixel×9-band multispectral image cubes. Each multispectral image cube therefore contains 1280 nine-band spectra. By averaging these across a region of interest drawn in the center of the image around the center of the fiber bundle, a mean spectrum was determined for each image (this occurs prior to reflectance normalization such that brighter pixels contribute more to the mean image spectrum). By averaging the spectra across images of the same tissue type within a given patient, mean “patient spectra” were determined. These different categories enabled us to test the performance of different classification methods on a per-pixel, per-image, or per-patient basis.

To quantify the variance in the data, standard deviations were calculated according to

$$\sigma =\sqrt{\left(\frac{\overset{9}{\underset{\lambda =1}{\sum }}\overset{n}{\underset{i=1}{\sum }}{\left(y_{i,\lambda }-\overset{¯}{y_{\lambda }}\right)}^{2}}{n}\right)},$$ (1)

where $y_{i,\lambda }$ is the reflectance i at band λ, with i=1:n, the number of spectra and λ=1:9, the nine bands of the spectrum, and $\overset{¯}{y_{\lambda }}$ is the mean reflectance in band λ. This was calculated within each group (e.g., per-patient) and the average over all groups (e.g., all patients) was calculated using a simple mean. For example, for the standard deviation per-image within-patients

$$\sigma_{\text{within}-\text{patient}}=\frac{\sum_{p=1}^{N}\sigma_{p}}{N},$$ (2)

where $\sigma_{p}$ is the standard deviation of all per-image spectra within patient p calculated according to Eq. (1) and N is the number of patients.

### 2.7 Classification of Multispectral Image Cubes to Discriminate Tissue Pathologies

For classification, the multispectral image cubes were randomly split into 80% for training and 20% for testing. Spectra calculated per-pixel and per-image were classified using seven methods commonly used in spectral data classification.^50^ Linear discriminant analysis (LDA) classifies spectra by finding a linear combination of features that maximizes the separation between classes relative to within-class variance in the feature space. K-nearest neighbor (KNN) algorithms classify spectra by choosing the most frequent classes of KNN data points in the feature space (k=3, 5, and 7 were tested). Both LDA and KNN classifiers were applied using the nine-band spectrum directly as a nine-element feature vector. While LDA assumes linear decision boundaries, the KNN algorithm is nonparametric so makes no assumptions about the shape of the decision boundaries. LDA also assumes variables are Gaussian distributed. The data were also projected onto principal component axes using principal component analysis (PCA) and the weights used for KNN classification. Spectral angle mapping (SAM) calculates the n-dimensional angle between a target spectrum and a reference spectrum; in this case, n=9. The reference spectra are the mean spectra per-pathology within the training dataset; thus three spectral angles are calculated for each target spectrum — $\theta_{\text{target-squamous}}$, $\theta_{\text{target-}\mathrm{NDBE}}$, and $\theta_{\text{target-neoplasia}}$. For a simple-SAM classification, the minimum of these three angles was taken as the predicted class. Alternatively, the angles were treated as a three-element feature vector and classified using LDA or KNN.

Finally, a neural network (NN) was used for classification. NNs perform classification by passing an input vector, in this case a nine-band spectrum, through a series of artificial neurons, with each neuron outputting some nonlinear function of its inputs with some weight that is adjusted during training. The output values of the final layer determine the classification. In contrast to LDA, NN classification does not make assumptions about the distribution of input data nor the shape of decision boundaries. The NN was implemented using a two-layer feedforward network, with a sigmoid transfer function in the hidden layer (10 neurons) and a linear transfer function in the output layer, using the MATLAB (MathWorks) Neural Network Pattern Recognition app. The 20% testing image cubes were randomly split into 50% for validation and 50% for testing (10% of all image cubes each). In summary, the seven classification methods compared were LDA, KNN, PCA-KNN, simple-SAM, SAM-LDA, SAM-KNN, and NN.

Accuracy for per-pixel classification was calculated in three ways: per-pixel accuracy, the percentage of pixels classified correctly over all pixels; per-image accuracy, the average percentage of pixels classified correctly in an image; and majority-pixel-per-image, the percentage of correctly classified images based on a relative majority decision (plurality) of all pixels within an image (if there is a tie between two classes, the image was counted as incorrectly classified). Accuracy for per-image-spectrum was the percentage of correctly classified mean-image-spectra.

## 3 Results

### 3.1 Clinical Study Recruitment

Between May 2018 and December 2019, a total of 20 subjects were recruited to this pilot clinical trial (Table S1 in Supplementary Material). All patients provided written informed consent. Of these, one subject was considered unfit for endoscopic procedure due to concomitant acute comorbidity and one was excluded as the visible lesion was too small for spectral imaging. Of the 18 subjects that underwent standard-of-care endoscopic procedures, three were excluded from analysis because of insufficient illumination for spectral imaging using the first prototype (n=2) and failure of the standard-of-care recording unit (n=1).

The multispectral endoscope was deployed to acquire *in vivo* esophageal image cubes. The study design prioritized the collection of image cubes matched to gold-standard diagnosis from histopathology (Fig. S1 in the Supplementary Material). To do this, cautery marks were made in two distinct regions of the esophagus, one deemed suspicious and one a control (Fig. S2A in Supplementary Material). The marked regions were inspected with the multispectral endoscope (Fig. S2B in the Supplementary Material), then the regions were biopsied (Fig. S2C in the Supplementary Material) and diagnosed based on the histopathologic analysis of the biopsies (Fig. S2D in the Supplementary Material).

Data were collected in 15 subjects at 44 distinct regions: 9 regions of squamous tissue, 24 regions of NDBE, and 11 regions of neoplasia. Of the 35 BE tissue regions, 34 were confirmed with histopathological assessment from 45 collected samples (33 biopsies, 14 endoscopic mucosal resections). In patient number 12, the control region identified on endoscopy was diagnosed as dysplastic by histopathology, so spectra that were incidentally captured from another region of NDBE were taken as NDBE without confirmation from histopathology. Coregistration between imaging data and histopathological diagnosis was successful for 30 regions (20 NDBE and 10 neoplasia), yielding a total of 570 labeled image cubes for analysis, plus an additional 157 image cubes from nine squamous regions (Table 1, Fig. S4 in the Supplementary Material). The mean exposure time was 200±50  ms. MSI lengthened the procedure time by <10  min per trial (data captured for mean of 5.2±1.5  min per trial).

**Table 1:** Summary of the collected image cubes and biopsies for matched regions.

Trial number	Barrett’s length (C = circumferential, *M* = maximum extent)/cm	Location/cm	Location/o’clock	Initial label during endoscopy	Tissue acquisition	Diagnosis from histopathology	Number of matched image cubes
3	C0M2	37	8	Suspicious	EMR × 1	NDBE	12
		37	4	Control	Biopsy × 1	NDBE	11
4	C4M6	36	6	Control	Biopsy × 1	NDBE	60
5	C5M7	31	9	Control	Biopsy × 1	NDBE	28
6	C11M14	31	9	Control	Biopsy × 1	NDBE	6
		28	6	Control	Biopsy × 1	NDBE	9
7	C11M12	35	9	Control	Biopsy × 2	NDBE	8
		32	5	Control	Biopsy × 2	NDBE	7
8	C6M7	32	12	Control	Biopsy × 1	NDBE	32
		30	6	Control	Biopsy × 1	NDBE	4
9		34	3	Suspicious	EMR × 1	IMC	9
	C5M7	33	3	Suspicious	EMR × 1	IMC	15
		30	9	Control	Biopsy × 1	NDBE	5
10	C14M14	32	10	Suspicious	Biopsy × 2	HGD	8
		24	10	Control	Biopsy × 1	NDBE	7
11	C2M5	35	12	Suspicious	EMR × 1	HGD	8
		36	6	Control	Biopsy × 1	NDBE	15
12	C2M4	41	6	Suspicious	EMR × 1	LGD	30
		39	8	Control	None	NA	5
		39	6	Control	Biopsy × 1	LGD	3
14	C0M3	36	3	Suspicious	Biopsy × 2	NDBE	8
		38	3	Control	Biopsy ×1	NDBE	7
15	C3M4	35 to 38	9	Suspicious	EMR × 4	IMC	11
		37	3	Control	Biopsy × 1	NDBE	8
16	C0M4	40	11	Suspicious	Biopsy × 1	IMC	7
		39	3	Control	Biopsy × 1	NDBE	4
17	C0M2	37	1	Suspicious	Biopsy × 2	NDBE	7
18	C5M7	26	12	Suspicious	EMR × 2	HGD	134
		25	9	Control	Biopsy × 1	NDBE	95
		26	3	Suspicious	EMR × 1	HGD	7

EMR, endoscopic mucosal resection; HGD, high grade dysplasia; IMC, intramucosal carcinoma; LGD, low grade dysplasia; NDBE, non-dysplastic Barrett’s esophagus; NR, not reported.

### 3.2 Machine Learning Enables Classification of Reference Color Spectra

**Fig. 4:**
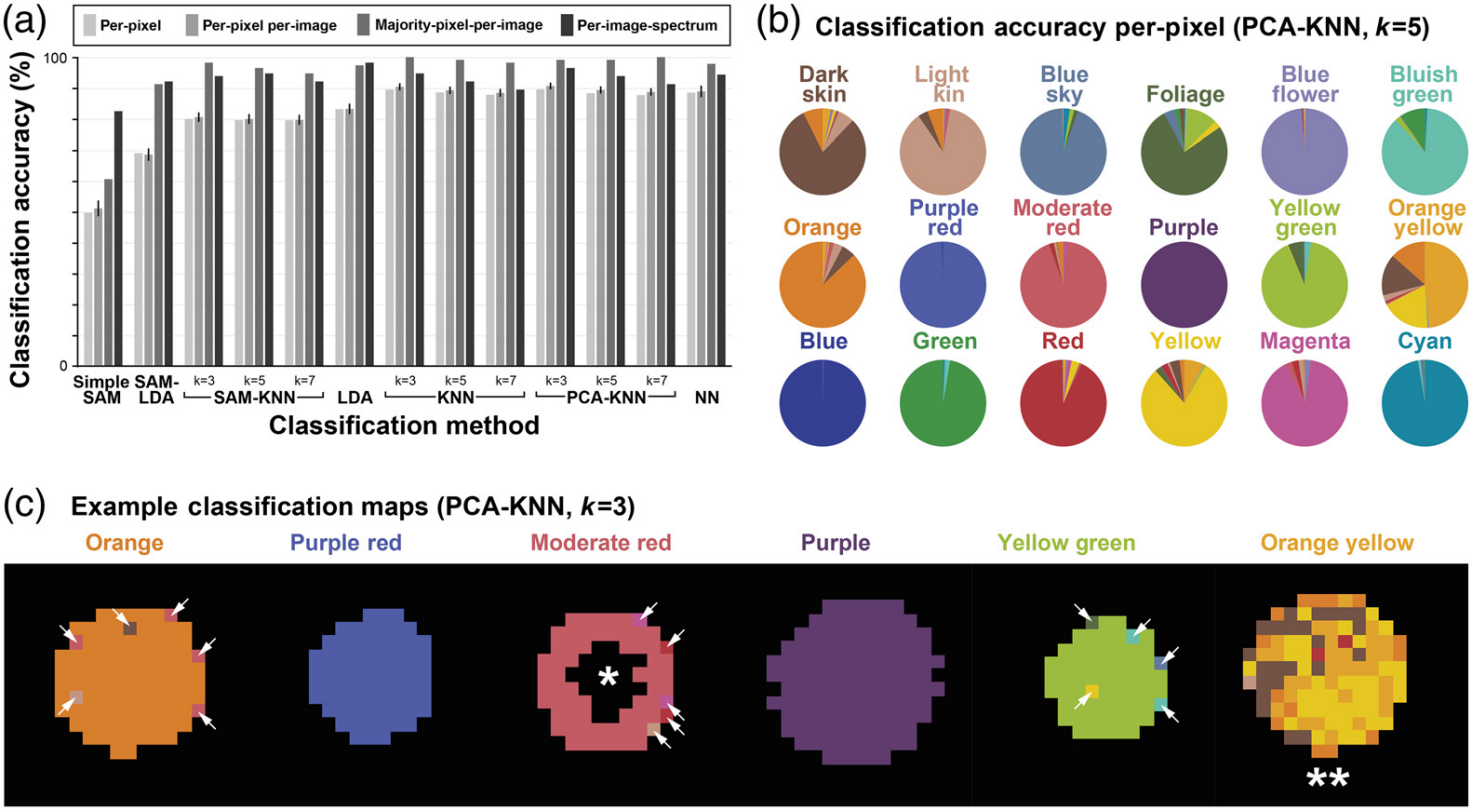
Machine learning enables classification of multispectral images of color chart. (a) Seven classification approaches were compared for classification of color chart image cubes. The best classification approach was PCA-KNN. (b) Per-pixel accuracies are shown as pie charts for each color in the Macbeth color chart. (c) Example classification maps are shown for 40-pixel×32-pixel×9-band image cubes of the middle color row of the Macbeth color chart. White arrows highlight misclassified pixels, which often lie close to the edge of the illuminated region. *The dark hole in the center of the “moderate red” image is a region of specular reflection. **Many of the “orange yellow” pixels are misclassified but are of a similar shade to the correct “orange yellow” color.

To validate the performance of the snapshot multispectral endoscope, handheld imaging of a Macbeth color chart was performed prior to imaging of four patients, resulting in 568 image cubes totaling 46,459 spectra for per-pixel classification [Figs. 3(a) and 3(b)]. The mean spectra per-pixel show distinct shapes but with significant overlap [Fig. 3(c)]. For classification, 454 image cubes (36,714 spectra) were used for training and 114 image cubes (9,745 spectra) were used for testing. Visualization of the training spectra by PCA not only shows clustering by color but also demonstrates the overlap between classes [Fig. 3(d)]. Despite this overlap, the classification was possible with 89.5% accuracy on a per-pixel basis (96.1±11.4% mean ± standard deviation per-image) and up to 99.1% when taking a majority decision per-image using PCA-KNN with k=3 [Fig. 4(a)]. On per-image-spectrum classification basis, the accuracy was 96.5%. As expected, per-pixel classification results for each color show that misclassification often occurred between similar shades [Fig. 4(b)] and at the edges of the illuminated area, where noise is more apparent [Fig. 4(c)]. Examples of classified image cubes representing colors from the middle row of the Macbeth color chart are shown in Fig. 4(c).

### 3.3 Spectra Show Promising Differences Between Tissue Types Despite Considerable Intra- and Interpatient Variation

**Fig. 5:**
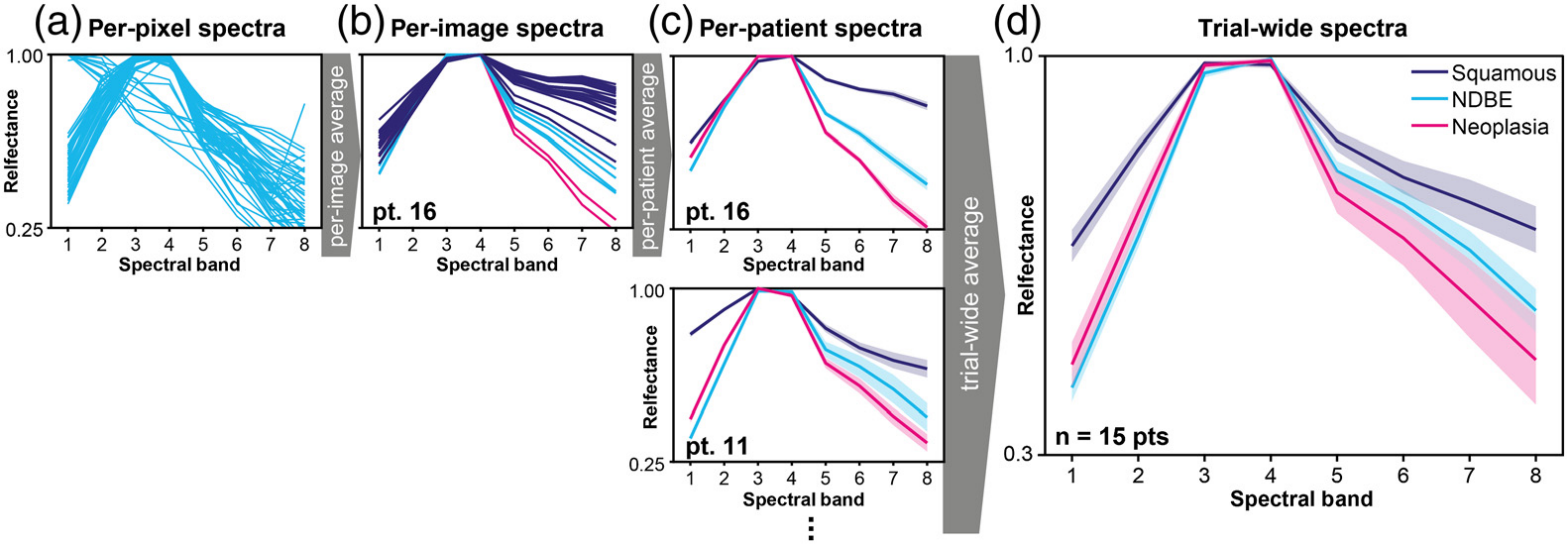
Spectra show promising differences between tissue types. (a) Per-pixel spectra for a single image of NDBE in patient 16. (b) All per-image spectra for patient 16. (c) Per-patient spectra for patient 16 and patient 11 are shown. The shaded region is the standard error from the standard across per-image spectra. (d) The trial-wide spectra. The shaded region is the standard error from the standard deviation across per-patient spectra.

**Fig. 6:**
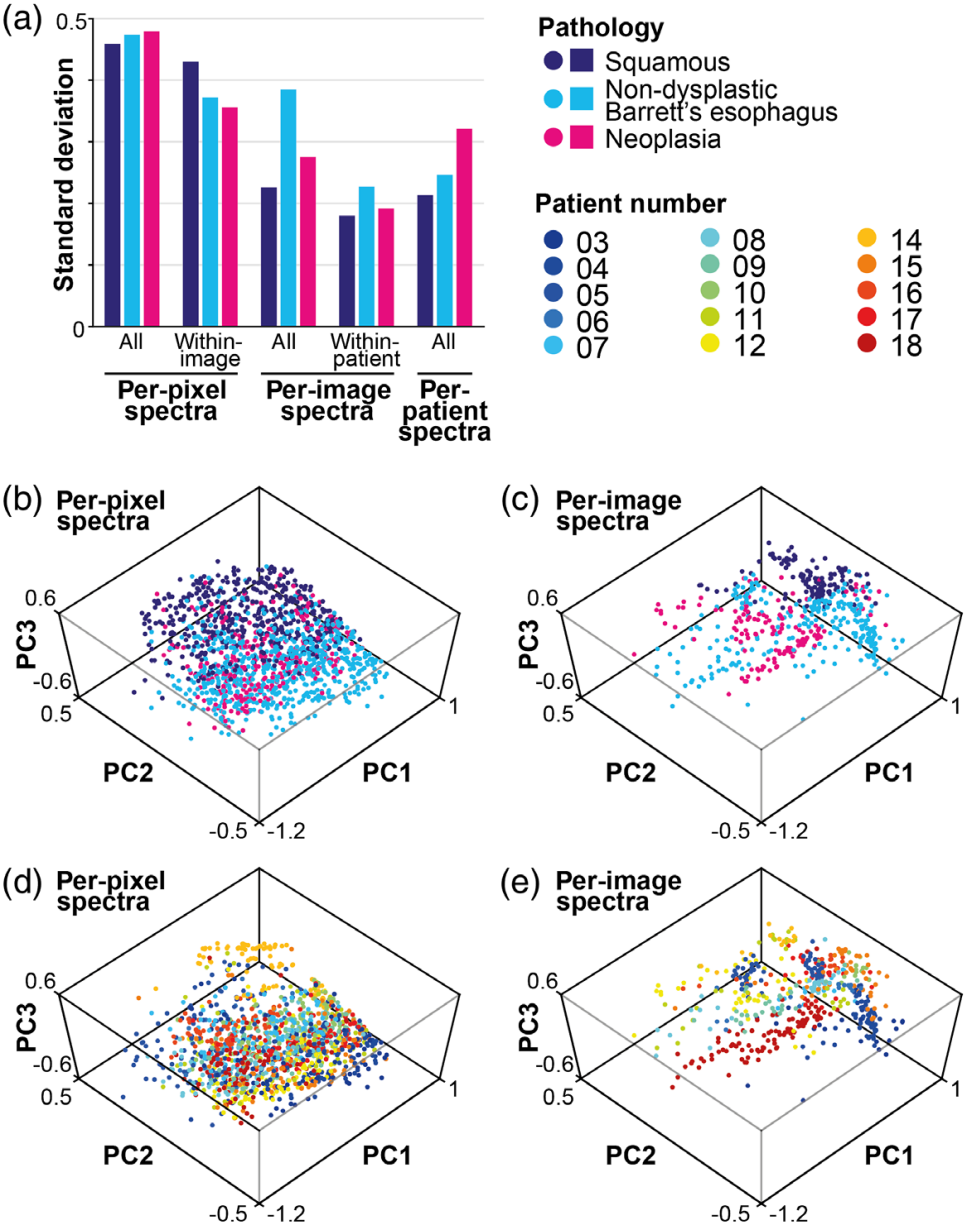
Spectra show considerable variance between patients. (a) The standard deviation is shown for per-pixel spectra, per-image spectra, and per-patient spectra for each pathology class. (b) PCA for per-pixel spectra shows clustering by pathology but with considerable overlap. (c) PCA of per-image spectra shows a more defined clustering per-pathology. Both (d) per-pixel spectra and (e) per-image spectra show visible clustering by patient, making clear the interpatient heterogeneity in the dataset.

We first evaluated the variance within our data. There is considerable variance among the spectra, per-pixel [Fig. 5(a)], per-image [Fig. 5(b)], and per-patient [Fig. 5(c)], but the average spectra across patients show clear differences among different histopathological categories [Fig. 5(d)]. We therefore sought to better understand the nature of the variance in the dataset (Fig. 6). The variance over all per-patient spectra, and over all per-pixel spectra, increases with progression of disease (0.213, 0.246, 0.321 for per-patient spectra and 0.459, 0.474, 0.479 for per-pixel spectra in regions of squamous, NDBE, and neoplasia, respectively). This is likely due to the increasing heterogeneity of the diseased tissues compared with healthy squamous tissue. Interestingly, the per-image variance within-patients is largest in NDBE. This might be due to intrapatient heterogeneity as multiple distinct regions of NDBE were imaged in four of the patients. It might also reflect the mosaic of cell types found in NDBE. The overall variance of spectra calculated per-image is smaller than the overall variance in the per-pixel spectra (0.226 versus 0.459, 0.385 versus 0.474, 0.276 versus 0.479 for squamous, NDBE, and neoplasia, respectively), perhaps due to noise removal by averaging in the images [Fig. 6(a)]. The same effect can be observed in PCA plots, where the spectra per-pixel [Fig. 6(b)] cluster less well than the spectral per-image [Fig. 6(c)]. Spectra per-pixel and spectra per-image cluster by pathology [Figs. 6(b) and 6(c)] and by patient [Figs. 6(d) and 6(e)], which suggests significant interpatient differences in the dataset, further supported by the standard deviation calculations [Fig. 6(a)]. The within-patient variance in per-image spectra is smaller than the total variance (0.180 versus 0.226, 0.227 versus 0.385, 0.192 versus 0.276 for squamous, NDBE, and neoplasia, respectively), suggesting there are notable interpatient differences, which would make generalizable classification challenging.

### 3.4 Snapshot Multispectral Endoscopy Shows Promising Accuracy for *In Vivo* Tissue Classification

**Fig. 7:**
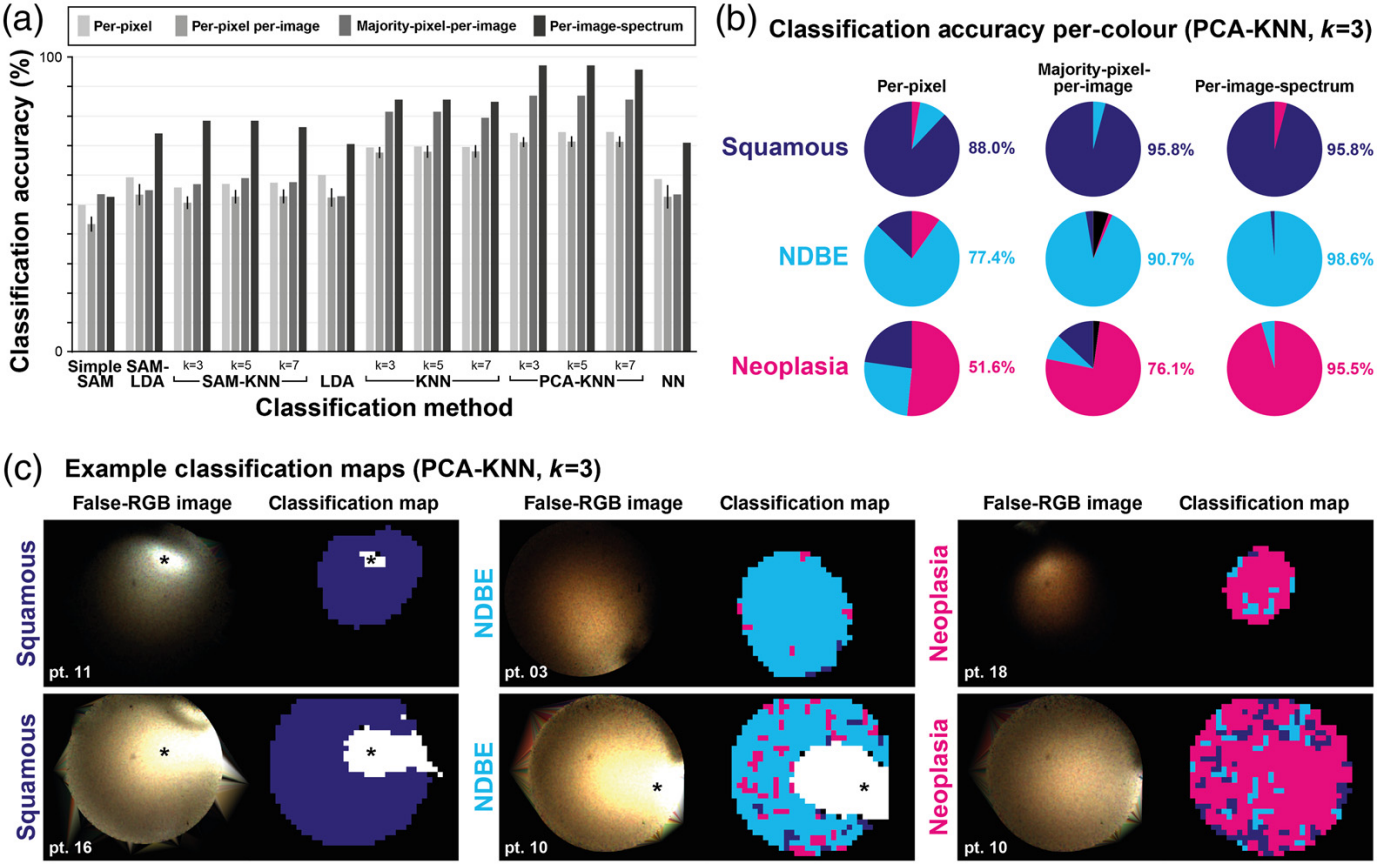
Snapshot multispectral endoscopy shows promising accuracy for *in vivo* tissue classification. (a) Seven approaches were compared for tissue classification. (b) The best classification approach was PCA-KNN with k=3. Per-pixel, majority-pixel-per-image, and per-image-spectrum accuracies are shown for each pathology. Black sectors represent images where majority pixel decision could not be reached. (c) Example classification maps of 40-pixel×32-pixel×9-band image cubes are shown for each pathology alongside the matched false-RGB image generated by assigning bands 2, 3, and broadband to R, G, and B colors, respectively. *Specular reflections are excluded from the classification and marked as white in the classification maps.

For classification, the patient data were split into training data comprising of 581 multispectral image cubes (121,600 spectra) and test data comprising of 146 image cubes (31,405 spectra). Seven classification methods were compared [Fig. 7(a)]. Despite the substantial aforementioned variance in the dataset, the classification per-pixel was possible with 74.2% accuracy (71.2±20.2% mean ± standard deviation per-image), rising to 87.0% accuracy when taking a majority pixel decision per-image using PCA-KNN with k=3 [Fig. 7(a)]. Per-image-spectrum classification increased accuracy to 96.5%. The classification results for each pathology show that squamous tissue was most reliably classified, whereas neoplasia was most difficult to classify [Fig. 7(b)]. This is in line with our expectations given the variance described in the dataset, as well as the familiar clinical challenge of distinguishing neoplasia. Nevertheless, on majority-pixel-per-image classification, accuracies of 95.8%, 90.7%, and 76.1% were achieved for squamous, NDBE, and neoplasia, respectively, and on per-image-spectrum classification, these rose to 95.8%, 98.6%, and 95.5%, respectively. Confusion matrices are shown alongside calculated values of sensitivity, specificity, positive predictive value, and negative predictive value for neoplasia classification in Fig. S5 and Table S2 in the Supplementary Material.

We then created classification maps representing the three pathologies [Fig. 7(c)] to display alongside RGB images, composed by assigning bands 3, 2 and 1 to red, green and blue, respectively. For visualization, map pixels whose four nearest-neighbor pixels are all a different class (to the said pixel) are defined as noise and replaced by the mode class of the four nearest-neighbor pixels. The maps illustrate how classification data could be presented to an operating endoscopist in real-time.

To calculate sensitivity, specificity, positive predictive value, and negative predictive value for detection of neoplasia in BE, the classification models were retrained for two-way classification using only NDBE and neoplastic image cubes (Fig. S6 and Table S3 in the Supplementary Material). For per-pixel classification PCA-KNN with k=3 achieved 60.7% sensitivity, 88.1% specificity, 71.7% positive predictive value, and 81.8% negative predictive value. In per-image-spectrum classification, this increased to 97.7% sensitivity, 100% specificity, 100% positive predictive values, and 98.6% negative predictive value.

## 4 Discussion

Advanced optical imaging modalities have potential to impact the care of patients with BE, by enhancing contrast for early lesions that can be treated using minimally invasive endoscopic therapy. MSI enables both spatial and spectral information to be captured during endoscopy, which has the potential to achieve this goal by revealing changes in the distribution of optical absorbers and scatterers between different disease states. Diffusely reflected light collected in our multispectral endoscope is estimated to penetrate approximately 200±100  μm into tissue,^51^ thus sampling the superficial mucosal layers where angiogenesis occurs in disease progression. We expect new microvessels to form from the pre-existing vascular network in the lamina propria and infiltrate the epithelium,^11^ increasing the hemoglobin abundance in the region sampled by our endoscope. Changes in cell and organelle morphology and arrangement might also contribute to changes in scattering in the epithelial layers.^52^^,^^53^

To test the potential of MSI, we constructed and tested in a pilot clinical study an SRDA-based snapshot multispectral endoscope capable of acquiring of nine-band multispectral image cubes *in vivo*. The SRDA-based snapshot multispectral endoscope was compatible with the clinical environment: it was deployed via the working channel of the standard-of-care endoscope allowing simultaneous standard-of-care imaging, illumination, articulation, insufflation, and washing capabilities; its deployment lengthened procedure time by <10  min (data captured for mean of 5.2±1.5  min per trial); it was compact (512  mm×480  mm footprint) and mobile, enabling it to be easily transported between procedure rooms between trials; and it was robust, allowing it to be used in a clinical setting with minimal realignment needed between trials.

Multispectral image cubes were successfully collected in 15 patients. Following MSI, biopsies were collected from the imaged regions, resulting in matched labeling from gold-standard diagnosis from histopathology. This resulted in the successful acquisition of 727 multispectral image cubes labeled with histopathological diagnosis from 39 distinct regions within 15 patients.

We found substantial intra- and interpatient variation, which is likely due to the intrinsic spatial heterogeneity of the disease. In regions with diagnosis of multifocal disease, it is likely that some acquired image cubes include contributions from both focal neoplasia and the surrounding “sea” of NDBE, which was not possible to mitigate in this study. The challenge of coregistering small-scale disease heterogeneity with *in vivo* imaging data has not yet been solved; as image segmentation becomes more detailed, so the coregistration of the image field with histopathology becomes more challenging. Careful consideration of these issues should be made in future studies aiming to use advanced spectral endoscopy methods to aid in BE surveillance. Additional variance could be attributed to variations in imaging geometry caused by peristalsis and the position of the lesion, though normalization should mitigate this effect. Nevertheless, the average spectra across patients showed clear differences between pathologies.

Seven classification methods were trained to classify the data as squamous tissue, NDBE, or neoplasia. PCA-KNN was found to provide the best performance in our dataset, classifying pathology with 74.2% accuracy per-pixel (71.2±20.2% mean ± standard deviation per-image), with majority pixel decision per-image achieving 87.0% accuracy and per-image-spectrum classification achieving 96.5% accuracy. Notably, two-way per-image-spectrum classification of neoplasia was possible with 99.1% accuracy (sensitivity 97.7%, specificity 100%, and negative predictive value 98.6%), which compares favorably with the American Society for Gastrointestinal Endoscopy Preservation and Incorporation of Valuable Endoscopic Innovations (PIVI)^54^ requirements for recommendation in BE surveillance^55^—per-patient sensitivity of ≥90%, negative predictive value ≥98%, and specificity ≥80% for detecting high-grade dysplasia or early esophageal adenocarcinoma—and with other emerging optical methods for endoscopic surveillance of BE.^8^ If these results are validated with a per-patient analysis in a larger patient cohort, multispectral endoscopy could be incorporated to clinical practice to improve the standard-of-care.

While these results are very promising, this first experience of applying an SRDA-based multispectral endoscope in patients revealed several limitations that will inform future work. First, our classification algorithms were tested using a random per-image split. Future work with a larger dataset should employ a per-patient split on a dedicated test set to assess the classification performance when dealing with unseen patients, particularly in light of the large interpatient variation seen in this trial.

Second, our system was constructed using a commercial SRDA, selected for the even distribution of spectral bands across the near-infrared window of biological tissue. Though our findings are promising, the sensor was not optimized for the detection of disease-specific spectral signatures and the nine-band spectra of NDBE and neoplasia showed significant overlap. The spectral bands available on the SRDA in future studies could be customized using recently reported fabrication processes^56^^,^^57^ and tailored to detection of specific disease signatures using spectral band optimization.^58^

A third limitation of our multispectral endoscope is the use of an imaging fiber bundle to carry light to detectors outside the body. Though this afforded us a swift route to clinical translation, it limits acquisition to 10,000 spatial points per image cube and consequently, images are of low quality in comparison to the high-definition images captured by standard of care endoscopes. In addition, attenuation due to the imaging fiber bundle also decreases sensitivity. This is worsened by the low sensitivity of the SRDA, partly due to the low quantum efficiency of the underlying sensor (<60%) and partly due to the low transmission of the deposited spectral filters (∼40%). For future studies and in the pathway to clinical adoption, the SRDA technology, customized as discussed above, would be most advantageous as a “chip-on-tip” device at the distal end of the endoscope, which would immediately overcome these limitations.

In summary, by combining the snapshot multispectral endoscope with machine learning techniques, we demonstrated a 74.2% accuracy per-pixel and 96.5% accuracy per-image for classifying squamous, NDBE, and neoplasia in a first-in-human trial. The next steps are to expand this work by testing custom SRDAs tailored to disease-specific spectral signatures. With further testing, multispectral endoscopy has the potential to improve detection of neoplasia during surveillance of BE.

## Code, Data, and Materials Availability

All code and data associated with this article are available through the University of Cambridge Data Repository at https://doi.org/10.17863/CAM.76523.
