# Supplementary material for: First-in-human pilot study of snapshot multispectral endoscopy for early detection of Barrett’s-related neoplasia
Source: J Biomed Opt. 2021 Oct 9;26(10):106002. doi: 10.1117/1.JBO.26.10.106002 (PMC8501416; doi:10.1117/1.JBO.26.10.106002)
Supplement: Supplementary file 1 [file JBO_026_106002_SD001.pdf]

Supplementary Material for:

First-in-human pilot study of snapshot multispectral endoscopy for early detection of Barrett's-related neoplasia

Exclusion Criteria

Exclusion criteria included history of esophageal stricture, pregnancy or breastfeeding, history of esophageal varices or liver impairment of moderate or worse severity (Child's Pugh class B or C), history of esophageal surgery except for uncomplicated fundoplication, history of coagulopathy (INR>1.3 and/or platelet count <75000) or on clopidogrel and/or anti-coagulant medication for high risk condition and unable to withhold medication temporarily.

Reflectance Correction

Note: for per-image colour chart classification, the white light reference was taken from the white patch on the Macbeth colour chart rather than the white reflectance tile.

Supplementary Figures and Tables

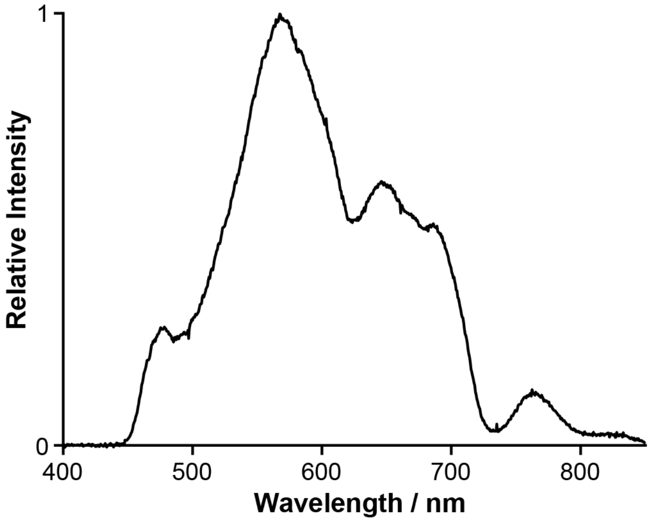

Supplementary Figure 1 | The spectrum of the multispectral endoscope illumination reflected from a white reflectance tile. This was measured by illuminating a diffusely reflecting white tile with the multispectral endoscope and focusing detected light from the imaging fiber bundle into a fiber coupled compact spectrometer (AvaSpec-ULS2048, spectral range 200–1,100 nm, grating 300 lines/mm, slit size 50 mm, Avantes).

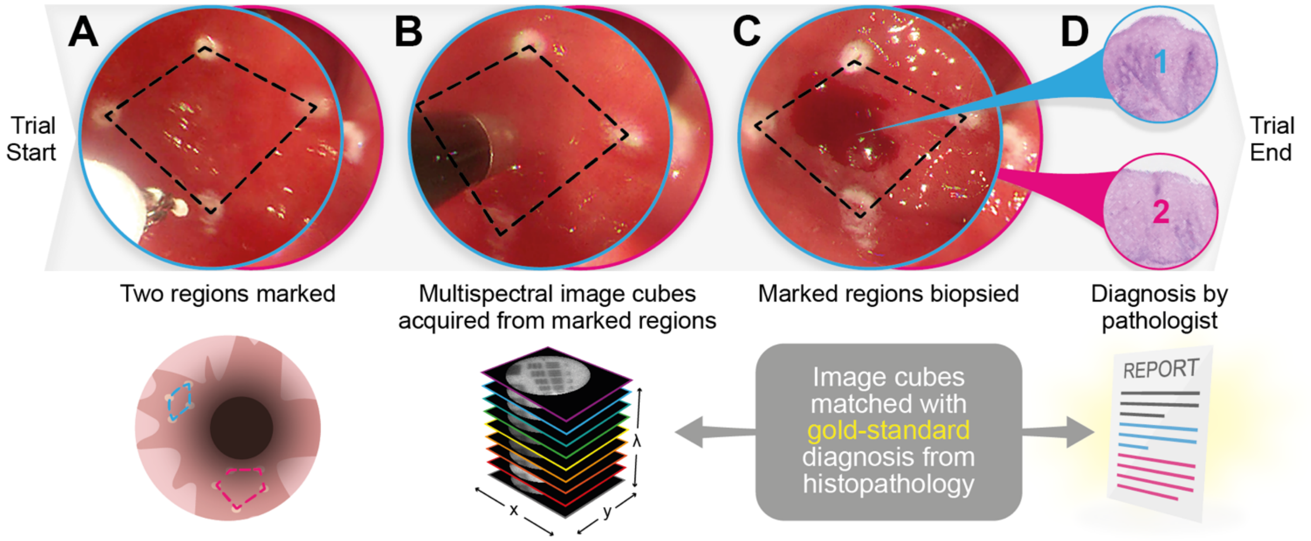

Supplementary Figure 2 | Overview of the trial protocol. (A) Two regions (*dashed lines*) are marked for inspection, one suspicious and one control, using cautery marking. **(B)** Multispectral images are acquired from each of the marked regions using the multispectral endoscope. **(C)** Following imaging, each of the marked regions is biopsied. **(D)** These biopsies are assessed by a histopathologist to yield a gold-standard diagnosis of each marked region. The acquired image cubes are thus matched with gold-standard diagnoses.

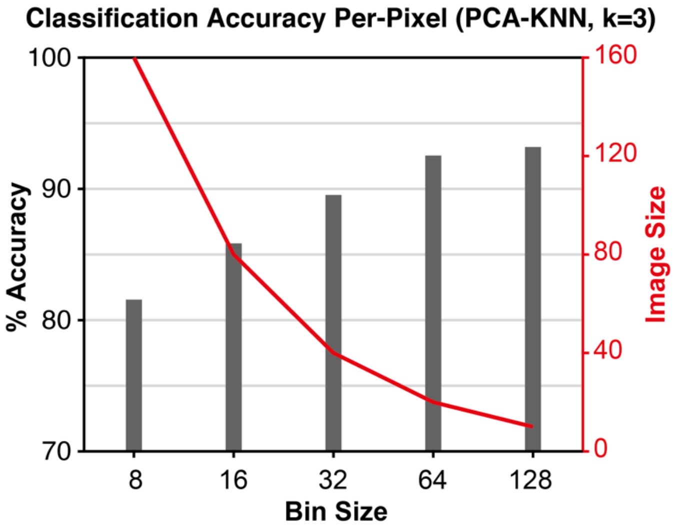

Supplementary Figure 3 | Classification accuracy versus image size for different bin sizes. Classification accuracy per-pixel for PCA-KNN classification of Macbeth color chart images (k=3) and image size (1280/bin size) are shown for bin sizes of 8, 16, 32, 64 and 128. A bin size of 32 was chosen as a compromise between accuracy and image size.

Supplementary Table 1 | Patient Demographics and Clinical Characteristics

Trial #	Trial Date	Collection of Matched Spectra and Histopathology	Comments	Sex	Age / years	Barrett's Length (C = Circumferential, M = Maximum Extent) / cm	Lesion Morphology (Paris Classification)
1	30/05/2018	N	Insufficient multispectral endoscope illumination	M	71	C3M7	0-IIb 5 mm
2	25/09/2018	N	Insufficient multispectral endoscope illumination	M	68	C3M5	0-IIb 5 mm
3	12/03/2019	Y		M	65	C0M2	0-IIb 5 mm
4	18/06/2019	Y		M	74	C4M6	0-IIa 10 mm
5	02/07/2019	Y		M	74	C5M7	no visible lesion
6	09/07/2019	Y		M	71	C11M14	no visible lesion
7	09/07/2019	Y		M	86	C11M12	no visible lesion
8	23/07/2019	Y		M	62	C6M7	no visible lesion
9	13/08/2019	Y		M	72	C5M7	0-IIb 15 mm
10	10/09/2019	Y		F	78	C14M14	0-IIb 10 mm
11	10/09/2019	Y		M	55	C2M5	0-IIb 4 mm
12	17/09/2019	Y		M	75	C2M4	0-IIb 5 mm
13	24/09/2019	N	Visible lesion too small for multispectral imaging	M	59	C1M5	0-IIb 1 mm
14	24/09/2019	Y		M	80	C0M3	0-IIb 2 mm
15	01/10/2019	Y		M	74	C3M4	0-IIb 25 mm
16	15/10/2019	Y		M	67	C0M4	0-IIa 20 mm
17	15/10/2019	Y		F	67	C0M2	0-IIb 2 mm
18	12/11/2019	Y		F	72	C5M7	0-IIb 20 mm
19	19/11/2019	N	Subject was considered unfit for endoscopic procedure due to concomitant acute comorbidity	M	NR	NA	NA
20	17/12/2019	N	SOC recording failed	M	81	C8M9	0-IIb 10 mm

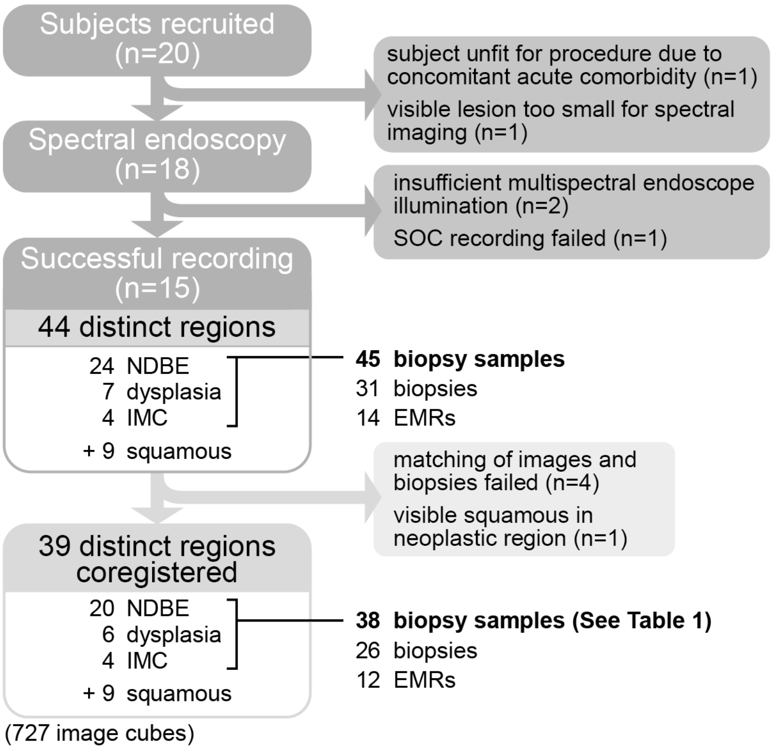

Supplementary Figure 4 | Recruitment Flow Chart

EMR, endoscopic mucosal resection; IMC, intramucosal carcinoma; NDBE, non-dysplastic Barrett's esophagus; SOC, standard of care.

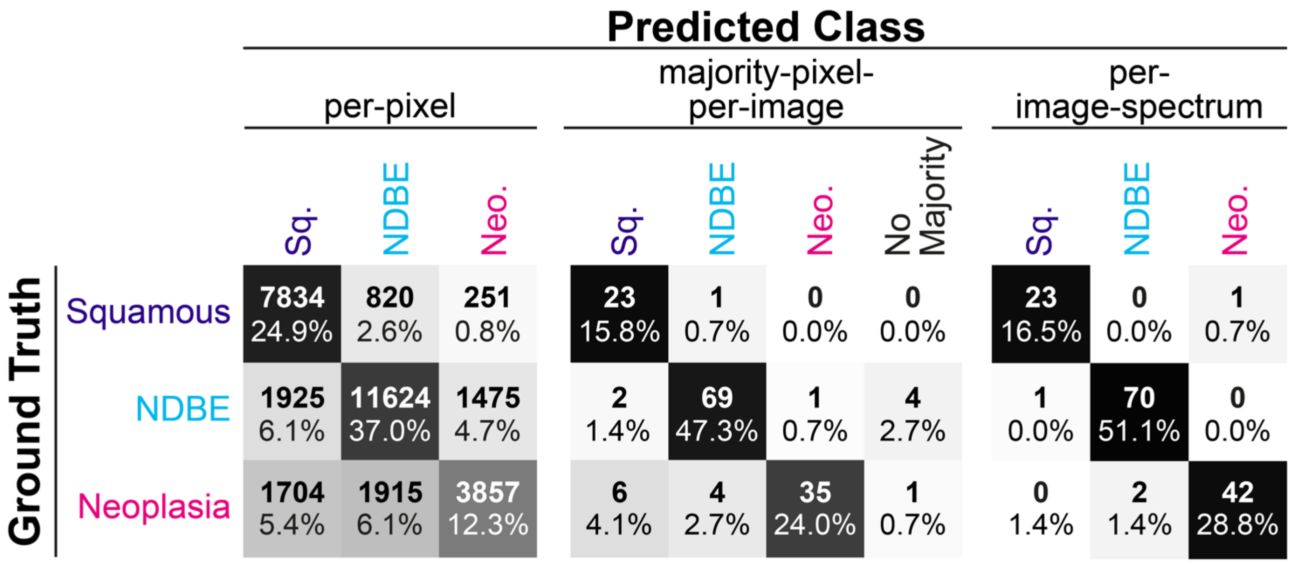

Supplementary Figure 5 | Confusion matrices for 3-way tissue classification using PCA-KNN with k=3

Supplementary Table 2 | Performance metrics for tissue classification using PCA-KNN with k=3

Comparison	Classification Performance				
	**Accuracy % (n/total)**	**Sensitivity % (n/total)**	**Specificity % (n/total)**	**Positive Predictive Value % (n/total)**	**Negative Predicative Value % (n/total)**
**per-pixel**					
**3-way Classification**					
Squamous	85.0 (26705/31405)	88.0 (7834/8905)	83.9 (18871/22500)	68.3 (7834/11463)	94.6 (18871/19942)
NDBE	80.5 (25270/31405)	77.4 (11624/15024)	83.3 (13646/16381)	81.0 (11624/14359)	80.1 (13646/17046)
Neoplasia	83.0 (26060/31405)	51.6 (3857/7476)	92.8 (22203/23929)	69.1 (3857/5583)	86.0 (22203/25822)
**2-way Classification***					
Neoplasia vs. NDBE	79.0 (17771/22500)	60.7 (4535/7476)	88.1 (13236/15024)	71.7 (4535/6323)	81.8 (13236/16177)
**majority-pixel-per-image**					
**3-way Classification**					
Squamous	93.8 (137/146)	95.8 (23/24)	93.4 (114/122)	74.2 (23/31)	99.1 (114/115)
NDBE	91.8 (134/146)	90.8 (69/76)	92.9 (65/70)	93.2 (69/74)	90.3 (65/72)
Neoplasia	91.8 (134/146)	76.1 (35/46)	99.0 (99/100)	97.2 (35/36)	90.0 (99/110)
**2-way Classification***					
Neoplasia vs. NDBE	92.6 (113/122)	80.4 (37/46)	100.0 (76/76)	100.0 (37/37)	89.4 (76/85)
**per-image-spectrum**					
**3-way Classification**					
Squamous	98.6 (137/139)	95.8 (23/24)	99.1 (114/115)	95.8 (23/24)	99.1 (114/115)
NDBE	97.8 (136/139)	98.6 (70/71)	97.1 (66/68)	97.2 (70/72)	98.5 (66/67)
Neoplasia	97.8 (136/139)	95.5 (42/44)	98.9 (94/95)	97.7 (42/43)	97.9 (94/96)
**2-way Classification***					
Neoplasia vs. NDBE	99.1 (114/115)	97.7 (43/44)	100.0 (71/71)	100.0 (43/43)	98.6 (71/72)

NDBE, non-dysplastic Barrett's esophagus.

* In 2-way comparisons, the class in *italics* is the target for purposes of classification performance metric.

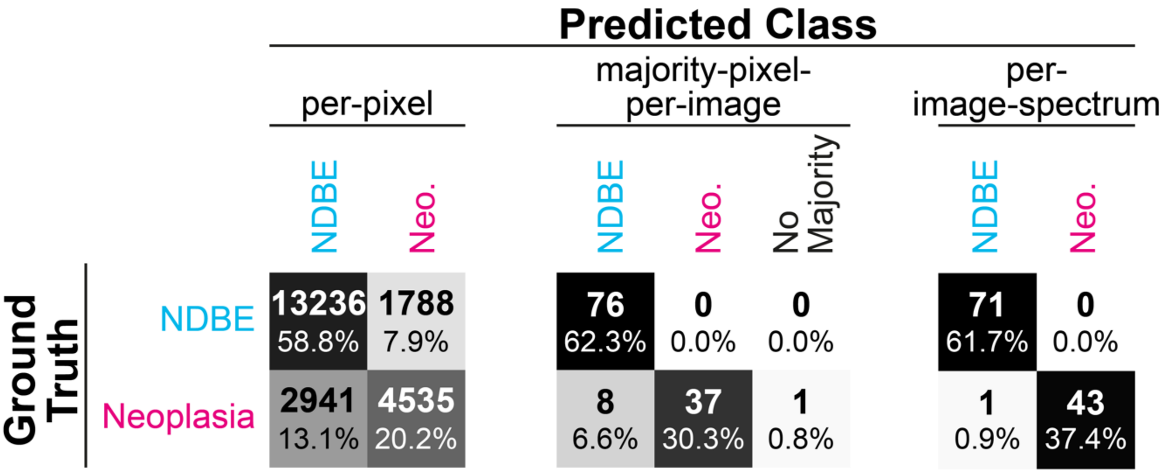

Supplementary Figure 6 | Confusion matrices for 2-way tissue classification using PCA-KNN with k=3
